# Editome Disease Knowledgebase v2.0: an updated resource of editome–disease associations through literature curation and integrative analysis

**DOI:** 10.1093/bioadv/vbaf012

**Published:** 2025-01-25

**Authors:** Tongtong Zhu, Yuan Chu, Guangyi Niu, Rong Pan, Ming Chen, Yuanyuan Cheng, Yuansheng Zhang, Zhao Li, Shuai Jiang, Lili Hao, Dong Zou, Tianyi Xu, Zhang Zhang

**Affiliations:** National Genomics Data Center, China National Center for Bioinformation, Beijing 100101, China; Beijing Institute of Genomics, Chinese Academy of Sciences, Beijing 100101, China; University of Chinese Academy of Sciences, Beijing 100049, China; National Genomics Data Center, China National Center for Bioinformation, Beijing 100101, China; Beijing Institute of Genomics, Chinese Academy of Sciences, Beijing 100101, China; University of Chinese Academy of Sciences, Beijing 100049, China; National Genomics Data Center, China National Center for Bioinformation, Beijing 100101, China; Beijing Institute of Genomics, Chinese Academy of Sciences, Beijing 100101, China; University of Chinese Academy of Sciences, Beijing 100049, China; National Genomics Data Center, China National Center for Bioinformation, Beijing 100101, China; Beijing Institute of Genomics, Chinese Academy of Sciences, Beijing 100101, China; University of Chinese Academy of Sciences, Beijing 100049, China; National Genomics Data Center, China National Center for Bioinformation, Beijing 100101, China; Beijing Institute of Genomics, Chinese Academy of Sciences, Beijing 100101, China; University of Chinese Academy of Sciences, Beijing 100049, China; National Genomics Data Center, China National Center for Bioinformation, Beijing 100101, China; Beijing Institute of Genomics, Chinese Academy of Sciences, Beijing 100101, China; University of Chinese Academy of Sciences, Beijing 100049, China; National Genomics Data Center, China National Center for Bioinformation, Beijing 100101, China; Beijing Institute of Genomics, Chinese Academy of Sciences, Beijing 100101, China; University of Chinese Academy of Sciences, Beijing 100049, China; National Genomics Data Center, China National Center for Bioinformation, Beijing 100101, China; Beijing Institute of Genomics, Chinese Academy of Sciences, Beijing 100101, China; University of Chinese Academy of Sciences, Beijing 100049, China; National Genomics Data Center, China National Center for Bioinformation, Beijing 100101, China; Beijing Institute of Genomics, Chinese Academy of Sciences, Beijing 100101, China; National Genomics Data Center, China National Center for Bioinformation, Beijing 100101, China; Beijing Institute of Genomics, Chinese Academy of Sciences, Beijing 100101, China; National Genomics Data Center, China National Center for Bioinformation, Beijing 100101, China; Beijing Institute of Genomics, Chinese Academy of Sciences, Beijing 100101, China; National Genomics Data Center, China National Center for Bioinformation, Beijing 100101, China; Beijing Institute of Genomics, Chinese Academy of Sciences, Beijing 100101, China; National Genomics Data Center, China National Center for Bioinformation, Beijing 100101, China; Beijing Institute of Genomics, Chinese Academy of Sciences, Beijing 100101, China; University of Chinese Academy of Sciences, Beijing 100049, China

## Abstract

**Motivation:**

Editome Disease Knowledgebase (EDK) is a curated resource of knowledge between RNA editome and human diseases. Since its first release in 2018, a number of studies have discovered previously uncharacterized editome–disease associations and generated an abundance of RNA editing datasets. Thus, it is desirable to make significant updates for EDK by incorporating more editome–disease associations as well as their related editing profiles.

**Results:**

Here, we present EDK v2.0, an updated version of editome–disease associations based on both literature curation and integrative analysis. EDK v2.0 incorporates a curated collection of 1097 editome–disease associations involving 115 diseases from 321 publications. Meanwhile, based on a standardized pipeline, EDK v2.0 provides RNA editing profiles from 48 datasets covering 2536 samples across 55 diseases. Through differential analysis on RNA editing, it further identifies a total of 7190 differential edited genes and 86 242 differential editing sites (DESs), leading to 266 339 DES–disease associations. Moreover, a curated list of 28 160 *cis*-RNA editing QTL associations, 458 187 DES–RNA binding protein associations, and 21 DES–RNA secondary structure associations are annotated and added to EDK v2.0. Additionally, it is equipped with a series of user-friendly tools to facilitate RNA editing online analysis.

**Availability and implementation:**

https://ngdc.cncb.ac.cn/edk/.

## 1 Introduction

RNA editing, as an important RNA modification that induces nucleotide alterations on endogenous and exogenous RNAs, plays a crucial role in different biological processes (Han *et al.* 2015). Multiple lines of evidence have been reported that RNA editing, mainly including adenosine-to-inosine (A-to-I; where inosine is subsequently recognized as guanosine) and cytidine-to-uridine (C-to-U) alteration types mediated by adenosine deaminases acting on RNA (ADAR) and apolipoprotein B mRNA editing catalytic polypeptide-like (APOBEC) enzymes, respectively, is associated closely with human diseases, covering nervous system diseases (Gaisler-Salomon *et al.* 2014), immune system diseases (Roth *et al.* 2018), viral infectious diseases (Zhu *et al.* 2023), and cancers (Chen *et al.* 2013). Additionally, to date, several studies have been conducted to yield RNA-editing-related landscapes from different aspects (Wu *et al.* 2023), such as RNA editing quantitative trait locus (edQTL) (Li *et al.* 2022), RNA binding protein (RBP) (Quinones-Valdez *et al.* 2019), and RNA secondary structure (RSS) (Wan *et al.* 2014), together providing fundamental annotations for studying human diseases.

Editome Disease Knowledgebase (EDK), an important resource for editome–disease associations officially released in 2018 (CNCB-NGDC Members and Partners 2024), has been established to provide a curated collection of 248 editome–disease associations involving 61 human diseases (Niu *et al.* 2019). Over the past several years, a number of studies powered by high-throughput sequencing technologies have discovered previously uncharacterized editome–disease associations and generated an abundance of RNA editing datasets. Moreover, differential edited genes (DEGs) and differential editing sites (DESs), which have been identified from these datasets yet through different bioinformatic pipelines, are critically important to help us decipher the pathogenesis of human diseases (Gabay *et al.* 2022, Li *et al.* 2022, Ruan *et al.* 2022, Zhou *et al.* 2023). Therefore, it is highly needed for EDK to incorporate more editome–disease associations through literature curation, as well as detect DEGs and DESs based on a standardized processing pipeline.

Here, we present an updated release of EDK v2.0 (https://ngdc.cncb.ac.cn/edk/) with significant updates and enhancements. Compared to the previous version, EDK v2.0 houses a total of 1097 editome–disease associations manually curated from 321 publications and identifies a number of DEGs and DESs from 48 datasets by utilizing a standardized processing pipeline. Based on this, it further provides a list of featured DESs that are specifically associated with edQTL, RBP, and RSS. Additionally, it is significantly enhanced by equipping with a series of tools.

## 2 Methods

### 2.1 Literature curation

To provide high-quality editome–disease associations, an improved curation model was built to meticulously curate editome–disease associations from RNA editing-related publications, which were retrieved in PubMed using keywords of “RNA editing” and “disease” (https://ngdc.cncb.ac.cn/edk/faq/). After manual curation, publications containing editome–disease associations with necessary descriptions on biological traits were incorporated in EDK v2.0. The curated editome–disease associations were further described into four sections: gene–disease associations, editing site–disease associations, virus–disease associations, and enzyme–disease associations. The curated biological trait entities were mapped to several resources, such as Disease Ontology (Schriml *et al.* 2022), GeneCard (Safran *et al.* 2010), and UniProt (Bateman *et al.* 2021).

### 2.2 Data integration and analysis

To identify A-to-I and C-to-U high-quality RNA editing sites in RNA-seq datasets, a standardized processing pipeline was built by REDItools (Lo Giudice *et al.* 2020). First, a list of RNA-seq datasets, characterized by median mapping rates ≥70%, was filtered from Sequence Read Archive (https://www.ncbi.nlm.nih.gov/sra/) for further processing. The metadata information of these datasets was obtained from Gene Expression Nebulas (Zhang *et al.* 2022). Second, GENToolkit computational pipeline was developed to obtain high-quality reads (https://ngdc.cncb.ac.cn/gen/toolkit/). The pipeline consists of the following steps: (i) Fastp (Chen *et al.* 2018) was used for trimming and filtering raw reads; (ii) HISAT2 (Pertea *et al.* 2016) was used to evaluate data quality, including mapping quality and coverage; and (iii) high-quality RNA-seq reads were aligned to the human reference genome by STAR alignment software (Dobin *et al.* 2013). Next, REDItools was applied to identify RNA editing sites. To minimize false positives of these sites, Pblat (Wang and Kong 2019) was employed to detect mismatched or multi-mapping reads, and samtools (Li *et al.* 2009) was used to remove duplicated reads. Additionally, the SNP file was downloaded from UCSC to filter SNP sites (http://hgdownload.soe.ucsc.edu/). Annotation of RNA editing site was retrieved from REDIportal (Picardi *et al.* 2017), DARNED (Ramaswami and Li 2014), and RepeatMasker (Tarailo-Graovac and Chen 2009). Gene annotation file was obtained from GENCODE v33 (Frankish *et al.* 2019).

Considering that quantitative analysis is unsuitable for comparative analysis across different datasets due to batch effects, we addressed the issue by defining the fold change score of RNA editing sites to qualitatively describe the difference between disease and healthy conditions. This approach accounts for sequencing discrepancies between different datasets. The fold change score is calculated by the ratio of the number of samples exhibiting RNA editing in disease to the number of samples exhibiting RNA editing in healthy condition:


(1) $$\mathrm{Fold}\;\mathrm{change}\;\mathrm{score}=\frac{\frac{{\mathrm{Edited}\;\mathrm{sample}}_{\mathrm{disease}}}{{\mathrm{Sample}}_{\mathrm{disease}}}}{\frac{{\mathrm{Edited}\;\mathrm{sample}}_{\mathrm{healthy}}}{{\mathrm{Sample}}_{\mathrm{healthy}}}},$$

where a fold change score of 0 indicates that the site was edited in healthy but not in disease condition, whereas a fold change score of infinity indicates that the site was edited in disease but not in healthy condition. To address the sparsity of RNA editing dataset, statistical analysis was used to examine the difference in disease and healthy conditions (Wilcoxon test, *P *<.05).

A list of featured DESs was summarized from genetic and transcriptional perspectives, including edQTL, RBP, and RSS. The list of edQTLs across various diseases/traits was obtained from a recent study (Li *et al.* 2022), the RNA binding target sequences of RBP were obtained from starBase/ENCORI database (Li *et al.* 2014), and the RSS affected by RNA editing were obtained from a study (Wan *et al.* 2014) and resources (Lin *et al.* 2020).

To investigate the impact of RNA editing on different diseases, we employed a hypergeometric test to calculate the disease risk score for each RNA editing site. The risk score for disease *j* at RNA editing site *i* is defined as:


(2) risk scorei, j=∑i=mMMiN-Mn-iNn,

where *N* is the total number of samples, *n* is the number of samples containing the RNA editing site, *M* is the number of patients, and *m* is the number of patients containing the RNA editing site.

### 2.3 Database implementation

EDK v2.0 was implemented using Spring Boot (https://spring.io/projects/spring-boot/) in conjunction with JPA as backend web framework. MySQL (https://www.mysql.com/) was used to serve as the database engine, providing robust backend support for data management and querying. HTML and CSS were utilized to construct the user interface. Semantic UI (https://semantic-ui.com/) was applied for enhancing the visual aesthetics of the web pages. JavaScript and AJAX technologies were employed to implement interactive features and asynchronous data loading. Highcharts (https://www.highcharts.com.cn/), ECharts (http://echarts.apache.org/), and DataTables (https://datatables.net/) were utilized for creating interactive data visualizations.

## 3 Results

EDK v2.0 is devoted to providing a comprehensive collection of high-quality editome–disease associations. Compared with the previous version, EDK v2.0 has been significantly updated in both data volume and web functionality (Table 1).

**Table 1.** Statistics of two versions of EDK.

Module	Item	v2.0	v1.0
Diseases	Disease categories	18	10
	Editome-associated diseases	154	61
	Diseases in literature curation	115^a^	61
	Diseases in integrative analysis	55^a^	/
Literature curation	Genes	86	49
	Editing sites	628	/
	Viruses	43	11
	Enzymes	11	6
	Publications	321	212
	Editome–disease associations	1097	248
	Gene–disease	110	/
	Editing site–disease	709	/
	Virus–disease	181	/
	Enzyme–disease	97	44
Integrative analysis	Editing datasets	48	/
	Samples	2536	/
	DEGs	7190	/
	DESs	86 242	/
	DES–disease associations	266 339	/
Featured annotation	edQTL associations	28 160	/
	DES–RBP associations	458 187	/
	DES–RSS associations	21	/
Tools	Editing site identification	Available	/
	Disease prediction	Available	/
	Gene–disease network construction	Available	/
	Cross-disease analysis	Available	/
	Editing site annotation	Available	/

a There are 16 diseases shared in both literature curation and integrative analysis.

**Figure 1.**
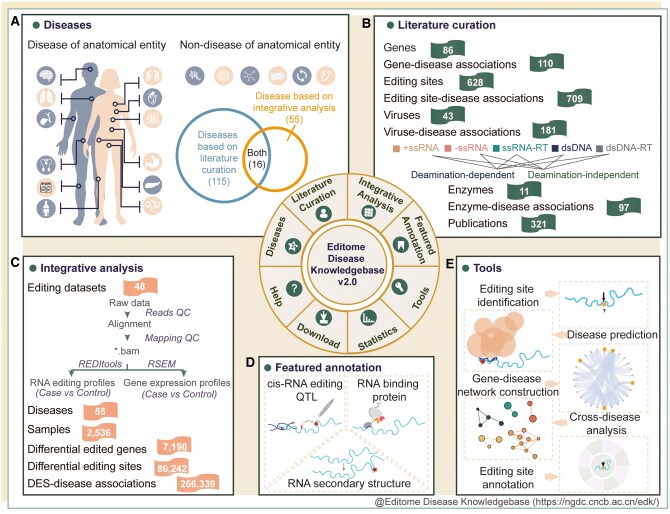
Schematic overview of EDK v2.0. (A) A standardized classification system that categorizes editome-associated diseases in EDK v2.0. (B) Statistics of gene-disease associations, editing site-disease associations, virus–disease associations, and enzyme–disease associations through literature curation in EDK v2.0. (C) Standardized data processing and integrative analysis results in EDK v2.0. (D) Comprehensive annotations of featured DESs, including edQTL, RBP, and RSS. (E) A series of online analysis tools.

Specifically, the number of curated editome–disease associations grows significantly from 248 to 1097 and accordingly the number of diseases increases from 61 to 115. Moreover, through differential analysis of RNA editing, a total of 7190 DEGs, 86 242 DESs, as well as 266 339 DES–disease associations computationally identified from 48 datasets, are newly added in EDK v2.0. A collection of 28 160 edQTL associations, 458 187 DES–RBP associations, and 21 DES–RSS associations are also identified and integrated. Additionally, a series of user-friendly tools is equipped to enhance the utility for editing site identification, disease prediction, gene–disease network construction, cross-disease analysis, and editing site annotation. All these information is well-managed and organized in terms of diseases, literature curation, integrative analysis, featured annotation, tools, statistics, download, and help (Fig. 1).

### 3.1 Expanded diseases associated with RNA editing

**Figure 2.**
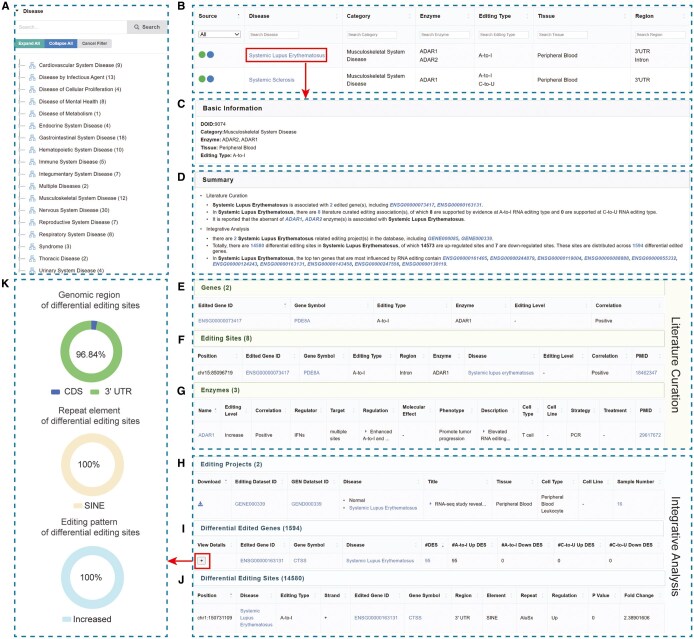
A screenshot of “disease” page in SLE. (A) Statistics of human diseases classified into 18 disease categories. (B) Overview of editome-associated diseases in EDK v2.0. (C) Basic information of SLE, such as DOID, disease category, aberrant enzyme, pathogenic tissue, RNA editing type, etc. (D) Summary information of SLE through literature curation and integrative analysis. (E–G) The editome–disease associations in SLE, including (E) gene–SLE associations, (F) editing site–SLE associations, and (G) enzyme–SLE associations. (H–K) The integrative analysis results, including (H) RNA editing datasets, (I) DEGs, (J) DESs, and (K) the distribution of genomic region, repeat element, and editing pattern.

EDK v2.0 houses 154 diseases that cover 18 disease categories (Fig. 2A), including 115 diseases manually curated from 321 publications and 55 diseases computationally identified from 48 datasets, which is more significantly enriched than the previous version containing 61 diseases in 10 disease categories. A list of all collected diseases as well as their related information, such as disease category, enzyme, editing type, tissue, and region, is provided (Fig. 2B). Among these diseases, 16 are supported by both literature curation and integrative analysis, namely, amyotrophic lateral sclerosis, autism spectrum disorder, breast cancer, chronic hepatitis, chronic lymphocytic leukemia, diabetes mellitus, hepatocellular carcinoma, melanoma, non-small-cell lung cancer, prostate cancer, psoriasis, rheumatoid arthritis, schizophrenia, sepsis, systemic lupus erythematosus (SLE), and systemic sclerosis (Fig. 1A). For each disease, EDK v2.0 sets up a dedicated disease page with four sections, including basic information, summary, literature curation, and integrative analysis (if available). Specifically, the basic information section provides a detailed description on the disease, such as DOID, disease category, disease description, etc. (Fig. 2C). The summary section is assigned to facilitate a quick understanding of associations between RNA editing and disease, along with hyperlinks to internal and external resources (Fig. 2D). To help users browse the disease-associated editome, EDK v2.0 provides a comprehensive characterization for each disease, including genes, editing sites, enzymes in the literature curation section, and incorporates DEGs and DESs in the integrative analysis section (Fig. 2E–J). Moreover, EDK v2.0 allows users to interactively access integrative analysis results by visualizing the distribution of genomic region, repeat element, and editing regulation (Fig. 2K).

### 3.2 Editome–disease associations based on literature curation

EDK v2.0 incorporates 1097 editome–disease associations that are manually curated from 321 publications. Specifically, it contains 110 gene–disease associations with 86 edited genes, 709 editing site–disease associations with 628 editing sites, 181 virus–disease associations with 43 viruses, and 97 enzyme–disease associations with 11 enzymes (Fig. 1B).

#### 3.2.1 Gene–disease associations

**Figure 3.**
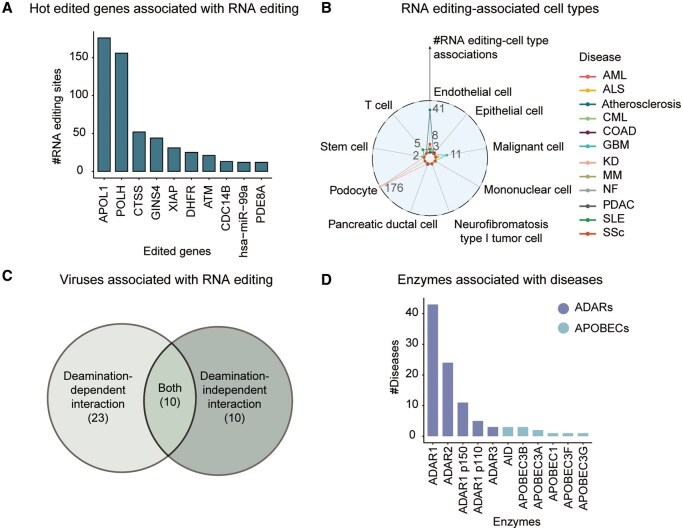
Statistics of editome–disease associations. (A) The top 10 hot edited genes as well as the number of corresponding RNA editing sites. (B) RNA editing site-associated cell types in different diseases. (C) Number of viruses associated with RNA editing in deamination-dependent and deamination-independent interactions. (D) Number of diseases affected by aberrant enzymes. AML, acute myeloid leukemia; ALS, amyotrophic lateral sclerosis; CML, chronic myeloid leukemia; COAD, colorectal cancer; GBM, glioblastoma; KD, kidney disease; MM, multiple myeloma; NF, neurofibromatosis; PDAC, pancreatic ductal adenocarcinoma; SSc, systemic sclerosis.

The 110 gene–disease associations cover 86 edited genes, namely, 60 mRNAs, 21 miRNAs, 4 circRNAs, and 1 lncRNA, in contrast to the previous version containing 49 genes that cover 32 mRNAs, 16 miRNAs, and 1 lncRNA. Among these genes, 39 genes have two or more aberrant sites, and representative examples include *CTSS*, *APOL1*, *POLH*, *GINS4*, *XIAP*, *DHFR*, *ATM*, *CDC14B*, *hsa-miR-99a*, and *PDE8A* (Fig. 3A). These genes are further characterized in terms of editing type (A-to-I, G-to-A, C-to-U, U-to-C, and G-to-C), enzyme (ADAR1, ADAR2, APOBEC, APOBEC3A, and APOBEC3B), editing pattern (increased, decreased, similar, present, and absent), and correlation (positive and negative). Furthermore, the same edited genes may influence the development of various diseases. For instance, aberrant sites in *CTSS* have been implicated in SLE, atherosclerosis, and systemic sclerosis (https://ngdc.cncb.ac.cn/edk/gene/CTSS/). Thus, EDK v2.0 assigns a gene page that contains a gene–disease network centered on this gene, so as to help researchers better understand the regulatory mechanisms for a specific edited gene and its associated disease(s).

#### 3.2.2 Site–disease associations

The 709 RNA editing site–disease associations correspond to 628 sites, viz., 517 in 3′UTR, 35 in intron, 32 in CDS, 28 in miRNA, 9 in exon, 3 in circRNA, 2 in 5′UTR, and 2 in lncRNA. Accordingly, the curation model of EDK v2.0 has been updated and enhanced with additional controlled terms and vocabularies to capture more important information (https://ngdc.cncb.ac.cn/edk/curation/sites/). For example, the term “editing pattern,” with controlled vocabularies (increased, decreased, similar, present, and absent), qualitatively describes the change of RNA editing levels in disease relative to healthy condition. The term “correlation” reflects the correlation between RNA editing site and disease. The term “molecular effect” represents the impact of RNA editing on the edited gene. Besides, two terms, “treatment” and “survival”, have been incorporated to enhance the utility of disease therapy. As RNA editing mechanisms can be better deciphered at the cellular level, therefore, we have meticulously curated RNA editing sites and their cellular information from related publications. The aberrant sites across various cell types are newly added, such as podocyte, endothelial cell, malignant cell, T cell, stem cell, mononuclear cell, pancreatic ductal cell, neurofibromatosis type I tumor cell, and epithelial cell (Fig. 3B). Collectively, these experimentally validated findings offer a higher-resolution understanding of RNA editing’s impact on cellular functions.

#### 3.2.3 Virus–disease associations

Host-mediated RNA editing exerts proviral or antiviral effects in various viruses and thereby associates with the pathogenesis of infectious diseases (Zhu *et al.* 2023). Therefore, EDK v2.0 encompasses a total of 181 virus–disease associations, spanning 43 viruses and 23 diseases, more significantly than the previous version with only 13 associations, 11 viruses, and 9 diseases. Moreover, these viruses are extended to five viral types, namely, positive-sense single-stranded RNA (+ssRNA), negative-sense single-stranded RNA (−ssRNA), reverse-transcribing single-stranded RNA (ssRNA-RT), double-stranded DNA (dsDNA), and reverse-transcribing double-stranded DNA (dsDNA-RT) viruses (Fig. 1B). More importantly, based on the interaction of RNA editing enzymes, EDK v2.0 classifies these viruses into two categories (Zhu *et al.* 2023): deamination-dependent and deamination-independent (Fig. 3C). In the deamination-dependent category, enzyme directly catalyzes the deamination of viral RNA substrates. For instance, in hepatitis delta virus, 18 associations have been identified around the hepatitis delta antigen (HDAg) sequence (https://ngdc.cncb.ac.cn/edk/virus/name/Hepatitis%20Delta%20Virus/). These aberrations result in the production of two variants of HDAg in hepatitis delta virus: HDAg-S and HDAg-L, which perform opposite functions (Hsu *et al.* 2019). In contrast, the deamination-independent category contains enzymes influencing viral activity through the indirect modulation of host immune response. Examples include those observed in hepatitis delta virus, BK polyomavirus, and Enterovirus 71 (https://ngdc.cncb.ac.cn/edk/curation/viruses/).

#### 3.2.4 Enzyme–disease associations

RNA editing enzymes, mainly including ADARs and APOBECs, play a pivotal role in RNA editing processes. Therefore, EDK v2.0 provides 97 aberrant enzyme–disease associations involving 11 enzymes, more enriched than the previous version that contains 44 associations and 6 enzymes (Fig. 3D). To further study the regulatory mechanisms between enzymes and diseases, these enzymes are further characterized in light of aberration (mutation, increased expression, decreased expression, RNA silence, and knockout), target information (target, regulation target, target function), correlation (positive and negative), regulator information (regulator and regulator function), phenotype, cell information (cell type and cell line), strategy, treatment, and PMID. Thus, a specific enzyme page is set up to structurally organize these items and ease users to browse associated diseases for a given enzyme. For example, it has been observed that mutation in *ADAR1* is positively correlated with Aicardi–Goutieres syndrome (Herbert 2020) and dyschromatosis symmetrica hereditarian (Suzuki *et al.* 2007). Additionally, expression change of *ADAR1* is associated with diseases, e.g. its increased expression in esophageal squamous cell carcinoma (Wu *et al.* 2021) and glioblastoma (Tassinari *et al.* 2021), and decreased expression in leukemia (Ma *et al.* 2011) (https://ngdc.cncb.ac.cn/edk/enzyme/ADAR1/).

### 3.3 Integrative analysis of RNA editing in diseases

EDK v2.0 features newly incorporation of A-to-I and C-to-U RNA editing profiles, DEGs, and DESs as well as their corresponding expression profiles, through integrative analysis based on 48 datasets encompassing 2536 samples across 55 diseases (Fig. 1C). Each dataset, covering at least one disease, is processed using a standardized pipeline and assigned with unique IDs for RNA editing profiles and gene expression profiles (https://ngdc.cncb.ac.cn/edk/datasets/projects/). To help users filter datasets, EDK v2.0 assigns a specific page for each dataset, involving dataset source, summary (strategy, species, tissue, and condition), sample information (basic information, sample characteristic, biological condition, experimental variables, protocol, sequencing, and assessing quality). Besides, for each dataset, DEG and DES are computationally identified through a unified pipeline. As a result, EDK v2.0 provides a comprehensive collection of 7190 DEGs and 86 242 DESs across 34 diseases, harboring 65 205 A-to-I and 21 037 C-to-U editing sites. Moreover, it offers additional information on these DESs, including basic information (position, associated disease, editing type, number of edited samples, etc.), annotation (edited gene, region, element, repeat, etc.), external link, and enzyme correlation. Additionally, due to far-reaching implications of RNA editing across various domains, including its associations with genomic variation (Park *et al.* 2021), RBP targets (Rahman *et al.* 2018), and RNA structure (Rieder and Reenan 2012), therefore, EDK v2.0 is committed to providing a list of high-quality functional evidence from edQTL, RBP, and RSS, which could be overviewed for featured DEGs and DESs (Fig. 1D). Additionally, the integrative analysis results across different diseases, including disease-specific DEGs, disease-common DEGs, disease-specific DESs, and disease-common DESs, are available at https://ngdc.cncb.ac.cn/edk/tool/crossdisease/.

### 3.4 Online analysis tools

To further enhance the usability of EDK v2.0 for studying RNA editing, a suite of user-friendly tools has been incorporated, including editing site identification, disease prediction, gene–disease network construction, cross-disease analysis, and editing site annotation (Fig. 1E). Editing site identification can help users to determine whether user-uploaded sites are RNA editing sites by leveraging extensive prior knowledge from multiple databases, such as REDIportal (Picardi *et al.* 2017) and DARNED (Kiran and Baranov 2010). Disease prediction is to assess the strength of relationship between a cluster of sites and specific diseases by calculating a list of predicted associations with hit ratio, average risk score, and max risk score. Recognizing that one edited gene may associate with multiple diseases, therefore, gene–disease network is provided to help users build a visualized network by linking various genes with multiple diseases. Cross-disease analysis performs comparative analysis based on DEGs and DESs among multiple diseases of interest to users and identifies disease-specific DEGs, disease-common DEGs, disease-specific DESs, and disease-common DESs. Editing site annotation provides a comprehensive summary of editing site annotations relevant to edQTL, RBP, and RSS.

### 3.5 Case study: RNA editing associated with SLE

As one of the systemic diseases, SLE usually presents stronger inflammatory responses and causes damage to organs throughout the human body. Studies have revealed the impact of genomic and transcriptomic variations on SLE, especially RNA editing (Bentham *et al.* 2015, Roth *et al.* 2018, Huang *et al.* 2021, Perez *et al.* 2022). Therefore, to demonstrate the utility of EDK v2.0, we elaborate on the associations between RNA editing and SLE as well as their corresponding genes, sites, and enzymes. Based on literature curation, we find that there are two edited genes (*CTSS* and *PDE8A*) in association with SLE, involving three sites located in *CTSS* and five located in *PDE8A*, respectively. The editing levels of these sites are enhanced and positively correlated with SLE (Orlowski *et al.* 2008, Vlachogiannis *et al.* 2021). Moreover, according to the current knowledge in EDK v2.0, there are 52 associations for *CTSS* and 12 associations for *PDE8A*, corresponding to 31 and 4 diseases, respectively. In addition, the aberrant enzymes are associated with SLE; the elevated RNA editing by *ADAR1* has been reported to be involved in the pathophysiology of SLE by increasing the autoantigen load (Laxminarayana *et al.* 2002, Roth *et al.* 2018). Also, altered *ADAR2* RNA editing contributes to the modulation of gene expression and immune functions in SLE patients (Laxminarayana *et al.* 2007).

**Figure 4.**
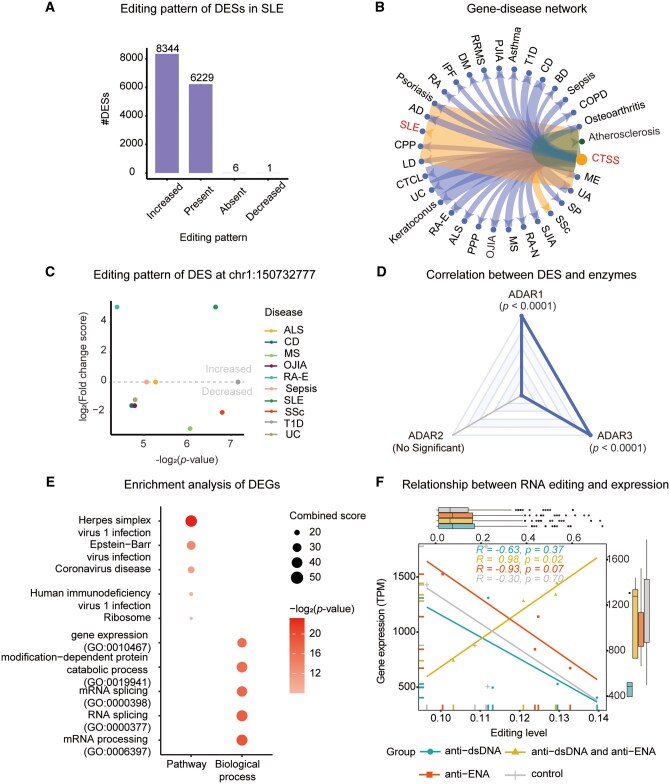
Integrative analysis results in SLE. (A) The editing pattern of DESs in SLE, including increased, present, absent, and decreased. (B) Gene–disease network between *CTSS* and diseases. Among them, the edited *CTSS* has been demonstrated to be associated with atherosclerosis through literature curation, as well as with SLE and SSc through both literature curation and integrative analysis. Width represents the number of associations between gene and disease. (C) Editing pattern of DES at chr1:150732777 on different diseases. (D) Relationship between RNA editing level at chr1:150732777 and expression value of ADARs in SLE. (E) Pathway and biological process enrichment analysis of DEGs in SLE by Enrichr. (F) Relationship between RNA editing level and expression value of corresponding edited *CTSS* gene in SLE. The four groups include patients with anti-dsDNA, anti-extractable nuclear antigens, both anti-dsDNA and anti-extractable nuclear antigens, and healthy individuals. Statistical *P*-values are calculated by Pearson’s correlation. ALS, amyotrophic lateral sclerosis; AD, atopic dermatitis; BD, bipolar disorder; COPD, chronic obstructive pulmonary disease; CPP, conventional plaque psoriasis; CD, Crohn’s disease; CTCL, cutaneous T-cell lymphoma; DM, diabetes mellitus; RA-E, established rheumatoid arthritis; IPF, idiopathic pulmonary fibrosis; LD, Lyme disease; MS, multiple sclerosis; ME, myalgic encephalomyelitis; RA-N, newly diagnosed rheumatoid arthritis; OJIA, oligoarticular juvenile idiopathic arthritis; PPP, palmoplantar psoriasis; PJIA, polyarticular juvenile idiopathic arthritis; RRMS, relapsing-remitting multiple sclerosis; RA, rheumatoid arthritis; SP, scalp psoriasis; SJIA, systemic juvenile idiopathic arthritis; SSc, systemic sclerosis; T1D, type 1 diabetes; UC, ulcerative colitis; UA, undifferentiated arthritis.

Through differential analysis on two SLE editing datasets, EDK v2.0 obtains 14 580 SLE-related DESs in 1594 DEGs. Among these DESs, there are, compared to healthy condition, 8344 with increased editing, 6229 present, 6 absent, and 1 decreased (Fig. 4A). The majority of these DESs (*n *=* *14 573, 99.95%) are found to be up-regulated in SLE compared to healthy condition, indicating that increased RNA editing is crucial for the pathogenesis of SLE. To compare the editing pattern of DESs among different diseases, EDK v2.0 has integrated a tabular table containing all DESs across different diseases (https://ngdc.cncb.ac.cn/edk/datasets/sites/). Taking *CTSS* as an example, there are 130 A-to-I DESs and 23 C-to-U DESs across 31 diseases, including SLE (Fig. 4B). The chr1:150732777 DES located in *CTSS*, according to the currently accumulated knowledge in EDK v2.0, is increased in SLE and rheumatoid arthritis and decreased in amyotrophic, Crohn’s disease, multiple sclerosis, oligoarticular juvenile idiopathic arthritis, sepsis, systemic sclerosis, type 1 diabetes, and ulcerative colitis (Fig. 4C). Moreover, according to our analysis and annotation, the site is situated in 3′UTR within the SINE/AluSz6 element and observed among 18 editing datasets with 346 (13.6%) samples. Within a range of 200 base pairs upstream and downstream of this site, there are 62 additional editing sites, indicating a hot editing region in *CTSS*. Meanwhile, there is a significant correlation between RNA editing level of this site and expression of *ADAR1* and *ADAR*3, indicating its potential role in RNA editing processes (Fig. 4D).

To further explore the relationship between enhanced RNA editing and SLE, functional enrichment analysis of these edited genes is performed. Our results show that these 1594 DEGs are significantly enriched in virus-related pathways, including herpes simplex virus 1 infection, Epstein–Barr virus infection, coronavirus disease, and human immunodeficiency virus 1 infection, which are all related to the immune response and inflammatory system (Fig. 4E). Meanwhile, these DEGs are also involved in the ribosome pathway, which is an important signaling pathway for cell fate determination. Moreover, we find that these edited genes participate in several important biological processes, including mRNA processing, RNA splicing, gene expression, and modification-dependent protein catabolic process (Fig. 4E). Additionally, we also analyze the impact of RNA editing on gene expression by calculating the correlation between editing level and gene expression in SLE. A significant correlation between RNA editing and expression of *CTSS* is observed in autoantibodies against group, such as anti-dsDNA and anti-extractable nuclear antigens (*R *=* *0.98, Fig. 4F). To sum up, as demonstrated by SLE, EDK v2.0 holds great promise in studying human diseases from transcriptome to editome.

## 4 Discussion

As one of the core resources of National Genomics Data Center, part of the China National Center for Bioinformation (CNCB-NGDC Members and Partners 2023, CNCB-NGDC Members and Partners 2024), EDK has been continuously evolving to keep up with the ever-growing knowledge and data in this area. It features literature curation to incorporate a high-quality collection of editome–disease associations. Furthermore, it highlights integrative analysis based on a unified pipeline to construct RNA editing profiles and gene expression profiles, and importantly, identify DEGs, DESs, and aberrant RNA editing associations across multiple diseases. Notably, in EDK v.2.0, a series of user-friendly tools is deployed to facilitate users to perform online analyses. Future plans are to integrate single-cell datasets, explore the impact of RNA editing on diseases at single-cell resolution, and establish close links with our internal databases, like Gene Expression Nebulas (Zhang *et al.* 2022) for gene expression datasets, Cell Taxonomy (Jiang *et al.* 2023) for cell type annotation, and Plant Editosome Database (Li *et al.* 2019) for plant RNA editing information. Taken together, EDK v2.0 bears great potential to serve as an important resource for decoding human diseases from the editome perspective.

## Data Availability

All data and analysis pipeline in EDK v2.0 are freely available online at https://ngdc.cncb.ac.cn/edk/.
